# Healthy vs. unhealthy food: a strategic choice for firms and consumers

**DOI:** 10.1186/2191-1991-1-4

**Published:** 2011-07-20

**Authors:** Fernando Antoñanzas, Roberto Rodríguez-Ibeas

**Affiliations:** 1 Department of Economics, University of La Rioja Cigüeña, 60, 26004-Logroño (Spain

**Keywords:** Healthy food, Unhealthy food, Obesity, Educational campaigns

## Abstract

In this paper, we carry out a theoretical analysis of the strategic choice made by firms regarding the type of food they market when they face consumers who care about the healthy/unhealthy attributes of the product but incur in emotional/health costs when the food they consume has unhealthy attributes. We consider a two-stage game. In the first stage, one of the firms chooses the unhealthy content of its product. In the second stage, both firms simultaneously decide their prices. We find that, depending on the parameters of the model, product differentiation can be maximal or less than maximal. The firm that produces the unhealthy food charges a higher price and obtains a larger share of the market unless the emotional/health costs and the unhealthy food production costs are relatively high. We also find that educational campaigns will not always reduce the demand for the unhealthy food or the degree of the unhealthy attribute.

**JEL Classification**:I10, I18, L11

## Background

A growing proportion of the food we eat is produced by industrial processes that combine a main ingredient with other ingredients to make the final product better looking and better tasting. Likewise, these procedures add value to the final product. For instance, flour is the main ingredient of bread, which for many generations was one of the basic foods for breakfast and snacks; however, bread has a relative low added value compared to croissants, donuts, cakes, cookies, brownies, and the like, which are nowadays commonly eaten. Flour is also an ingredient of these products although its proportion is lower than that found in plain bread. As a result, we eat more sugar and fat than needed, which are considered major causes of obesity.

Obesity trends are clearly upwards over the world. In the US, the percentage of people who are overweight is near 40% for men and around 30% for women [1]. In Europe the situation is a little bit better regarding the rates but the trend is similar to the US. For instance, in Spain, obesity rates are around 20% for the population over 25 years [2] and, in some regions, the percentage of people who are overweight is around 37% [3]. Caused by either the lack of calories consumption or the excess of calories intake, these obesity growing rates have many causes; some authors have analysed them from an economic point of view. As summarised by [4], the neoclassical economic point of view highlights several issues: the relative low price of food as compared to other consumption goods, the increase in the costs of calorie expenditure (the sedentary lifestyle of consumers) and the addictive features of unhealthy food. Further, it has been recently tested that declining food prices is one likely cause of the obesity epidemic [5]. Less evidence backs up the more controversial hypothesis that relative prices of healthy-unhealthy food have direct consequences on obesity. (See, for instance, [6]). In addition to the neoclassical arguments, other authors have focused on the behavioral theory of weight, pointing out that self-control and time-inconsistent preferences constitute the main explanation for obesity [7].

In spite of growing empirical economic literature dealing with obesity, theoretical analysis of decisions concerning the type of product (healthy or unhealthy) that firms sell in the market has received less attention. This choice depends, among other factors, on consumers' preferences and production costs. Regarding the latter, *a priori*, it can be assumed that the production costs of healthy goods are relatively low. Products traditionally considered healthy (for example, bread or plain cookies with no or low fat, sugar and salt content) are usually produced with simple industrial processes, and its production costs are indeed rather low. However, there are products with low levels of preservatives that lately have been labelled as healthy (organic, ecological and functional food) and whose production entails higher costs. The same distinction regarding production costs can be applied to unhealthy products. Thus, production of unhealthy food can be more expensive than that of healthy food (a cookie and a simple biscuit) or viceversa (ecological food versus non-ecological food).

On the demand side, some consumers are concerned about the proportion of unhealthy food, mainly products associated with obesity and cardiovascular diseases, that they eat. They read the labels of the industrially processed foods when available and incorporate this information into their decisions. However, other consumers value the external physical characteristics of food such as form, colour, smell, and taste, and disregard the further health consequences of their diet. More precisely, goods have several attributes from which consumers derive utility. In the case of food, the nutritional value is one of the main ones, some intrinsic attributes are directly linked to other hedonistic issues such as taste, the way that food was obtained and produced, as well as the healthy/unhealthy ingredients that the product contains. Further, individuals derive utility from other societal aspects related to the consumption of food such as belonging to a group of socially conscious people that is concerned with healthy food, the sustainability of the environment and its long-term health; consequently they invest in health by eating purely healthy products that may or may not be visually attractive or taste good. That is to say, consumers value additional societal attributes of the products. Further, some unhealthy products have two kind of effects: on the one hand they produce utility derived from their taste while eating them but on the other, they also have immediate negative consequences resulting from their consumption (long digestion, stomachache, dizziness, etc.).

Conventional utility functions that only incorporate satisfaction derived from physical attributes of goods are not an accurate description of what really matters to consumers. In this sense, neuroeconomics could help to better describe consumer behaviour [8,9]. For instance, a utility function that summarises satisfaction derived from the consumption of food could contain elements from the biological response to physical hunger, elements related to the pleasure of eating something that appeals to taste (intangible or hedonistic) and also elements related to sociological aspects (again intangible) such as remorse for eating unhealthy food or food contrary to social habits. Some of these elements are taken into account by individuals when they make their consumption decisions. and may have important consequences for the type of food produced by firms, in their market shares and in the prices. At the same time, firms that intend to satisfy individual needs know these elements, and have to make a strategic choice regarding the type of food they produce. Firms will try to differentiate their products by choosing features (either healthy or unhealthy) with the aim of gaining market share and increasing their profits.

In this paper, we consider a stylized model of vertical differentiation applied to food. In particular, we analyze a duopolistic market in which firms must decide the type of food they produce. One of the firms produces a healthy food without unhealthy attributes while the other firm makes decisions about the degree of unhealthy attributes in its product. We have chosen to model the decision made by firms that differentiate their products by adding a tasteful and unhealthy component such as fat or sugar instead of focusing on the decision of "healthy" firms. This choice is based on both the casual observation of the real world where the supply of food containing unhealthy components has grown enormously and on the fact that the first historical differentiation in food products came by adding them these components. On the demand side, we assume that consumers value the unhealthy attribute but, as mentioned above, they have some emotional/health costs derived from the consumption of the unhealthy product. Utility functions with arguments not directly related to the pleasure of consumption are also used in environmental product differentiation models [10,11].

We model the relationship between the firms as a two-stage game. In the first stage, one of the firms makes choices about the unhealthy content of its product. In the second, stage, both firms simultaneously decide their prices. We characterize the optimal degree of the unhealthy attribute as well as the equilibrium prices. Finally, we carry out a welfare analysis and suggest some public policies that could be implemented.

## Methods

We consider a standard duopolistic model of vertical product differentiation.^(*a*) ^Firm *i *produces a good (food) with healthy *h_i _*and unhealthy *s_i _*attributes and sells it at price *p_i_*, *i *∈ {1, 2}. Products of both firms have the same healthy attribute, *h*_1 _= *h*_2 _= *h*, but only firm 2 produces a good with unhealthy attribute: *s*_1 _= 0 and *s*_2 _= *s*, where *s *∈ [0, 1]. For the sake of simplicity and without loss of generality, we will assume that *h *= 1. Firm 2 can produce the unhealthy attribute *s *at a cost *C*(*s*), with *C*(0) = 0, *C*'(*s*) > 0, *C*"(*s*) > 0 and *C*'(0) = 0. To simplify the analysis, we assume that marginal production costs for both firms are zero.

There is a continuum of consumers indexed by *θ*, which is uniformly distributed in the interval [0, 1] with density one. The parameter *θ *measures the degree of emotional guilt derived from consuming a good with unhealthy attributes. Alternatively, it can be thought that *θ *denotes individual characteristics reflecting how unhealthy attributes affect consumer's health. All consumers obtain a utility of 1 if they consume the good produced by firm 1. The utility derived from consuming the good of firm 2 is given by 1 + *s - θd*, where *d *∈ [0, 1] denotes the emotional or the health-related-quality-of-life impact on utility. Consumers value the unhealthy attribute (e.g. the product tastes better), but, at the same time, they feel guilty for consuming a good with a higher unhealthy attribute. Thus, consumers differ in their feelings of guilt. Otherwise, they are identical, and their reservation prices equal to 1 and 1 + *s *for the goods of firms 1 and 2. Consumers buy as long as their net utility is non-negative. Each consumer is assumed to buy, at most, one unit of the product. There is a health cost *ls*, with *l *∈ [0, 1], per each consumed unit of the unhealthy product. Consumers internalize the emotional or the health-related-quality-of-life impact on utility when they made their consumption decisions. However, they do not take into account the health costs derived from unhealthy consumption borne by the public health system.

We consider a two-stage game. In the first stage, firm 2 decides the level of the unhealthy attribute *s*. In the second stage, the firms set simultaneously their prices. We solve the game by backward induction, and find the subgame perfect equilibrium. Our goal is to characterize the equilibrium prices and the optimal level of the unhealthy attribute.

### The firms' demand functions

Given (*p*_1_, *p*_2_), the consumer indifferent to both goods has a degree of guilt  that satisfies:

Therefore, . When , firm 1 sells to consumers with high degree of guilt while firm 2 sells to consumers with a degree of guilt below . When , no consumers patronize firm 1. When , all consumers buy from firm 1. Notice that consumers buy from firm 2 if and only if 1+*s -θd - p*_2 _≥ 0, e.g. if and only if , where . As *p*_1 _≤ 1, it follows that . ^(*b*)^

Firm 1's demand is given by:

Firm 2's demand is given by:

With the demand functions for both firms defined, we focus now on finding the equilibrium prices. In order to do that, we first characterise the firms' reaction functions.

### The reaction function of firm 1

Given *p*_2 _and *s*, firm 1 selects the price *p*_1 _to maximize its profits. Let *p*_2 _∈(*s - d*, *s*]. In this case, firm 1's best response is the solution to the following problem:

It is easy to verify that the solution to this problem is given by *p*_1 _= *f *(*p*_2_) = 0.5(*d *+ *p*_2 _- *s*). Let *p*_2 _≤ *s - d*. In this case, for all *p*_1_, firm 1's demand is zero.

Finally, let *p*_2 _>*s*. Firm 1 may choose *p*_1 _= *f *(*p*_2_) = *p*_2 _- *s *and serve the whole market. Alternatively, it can select a higher price, facing a demand of . In this case, it solves:

The solution to this problem is *p*_1 _= *f *(*p*_2_) = 0.5(*d *+ *p*_2 _- *s*) as long as *p*_2 _<*s *+ *d*. Thus, for *p*_2 _∈ (*s*, *s *+ *d*), we have two candidates for firm 1's best response. It can be easily shown that firm 1's profits are higher when it chooses *p*_1 _= *f*(*p*_2_) = 0.5(*d *+ *p*_2 _- *s*). When *p*_2 _≥ *s *+ *d*, firm 1's best response is *p*_1 _= *f *(*p*_2_) = *p*_2 _- *s*. Thus, the firm 1's reaction function is:(1)

Notice that when *s - d >*0, there are feasible prices for firm 2 (*p*_2 _≤ *s - d*) such that firm 1, regardless of its price, sells nothing. When *s - d *≤ 0, firm 1's best response is such that its demand is strictly positive for all feasible *p*_2_.

### The reaction function of firm 2

Given *p*_1 _and *s*, firm 2 selects the price *p*_2 _to maximize its profits. Let *d - s *≥ 0 and consider *p*_1 _>*d *- *s*. Firm 2 may choose *p*_2 _= *g*(*p*_1_) = *p*_1 _+ *s - d *and serve the whole market. Alternatively, it can select a higher price, facing a demand of . In this case, it solves:

The solution to this problem is *p*_2 _= *g*(*p*_1_) = 0.5(*s*+*p*_1_) as long as *p*_1 _< 2*d - s*. Thus, for *p*_1 _∈ (*d - s*, 2*d - s*), we have two candidates for firm 2's best response. It is straightforward to show that firm 2's profits are higher when *p*_2 _= *g*(*p*_1_) = 0.5(*s *+ *p*_1_). When *p*_1 _≥ 2*d - s*, firm 2's best response is *p*_2 _= *g *(*p*_1_) = *p*_1 _+ *s - d*. When *p*_1 _≤ *d - s*, firm 2's best response is *p*_2 _= *g*(*p*_1_) = 0.5(*s *+ *p*_1_). Thus, firm 2's reaction function when *d - s *≥ 0 is given by:(2)

Let *d - s <*0. If 2*d - s *≤ 0, firm 2's reaction function is given by *p*_2 _= *g*(*p*_1_) = *p*_1 _+ *s - d *for all *p*_1_. If 2*d - s >*0, firm 2's reaction function is given by (2).

## Results and discussion

### The equilibrium prices

A pair of prices  is an equilibrium in the pricing subgame if and only if  and .

Let 2*d - s *≤ 0. From (2), firm 2's best response is *p*_2 _= *g*(*p*_1_, *s*, *d*) = *p*_1 _+ *s - d *∀*p*_1 _and the equilibrium price of firm 2 is *s - d*. Firm 1 does not sell anything. ^(*c*)^

Let 2*d - s >*0. Notice that two possible situations can happen: either *d *≥ *s *or *d < s*. It can be shown that, regardless of the relationship between *d *and *s*, the equilibrium of the game is given by:

It can be easily checked from (1) and (2) that  and .

**Proposition 1 ***Let *2*d - s *≤ 0. *In equilibrium*, *firm *2 *chooses a price p*_2 _= *s - d*, *and firm *1 *sells nothing*. *Let *2*d - s >*0. *The equilibrium prices are **and *.

When the level of the unhealthy attribute is relatively high compared to *d *(*s *≥ 2*d*), product differentiation is so big that, in equilibrium, all consumers prefer to patronize firm 2. The highest net utility consumers can obtain from consuming the good from firm 1 is 1. That will happen if *p*_1 _= 0. However, in equilibrium, all consumers obtain a level of net utility above 1 when they patronize firm 2. Thus, firm 1 sells nothing. When the level of the unhealthy attribute is relatively low compared to *d *(s < 2*d*), product differentiation is so small that, in equilibrium, both firms share the market. Consumers with high degrees of emotional guilt find it profitable to patronize firm 1.

The demands in equilibrium are given by:(3)(4)

It is easy to see that firm 1's demand lowers with *s *and firm 2's demand rises with *s*. The higher the product differentiation, the more firm 2 sells. Also, firm 1's demand increases with *d *while firm 2's demand decreases with *d*. From Proposition 1 and the equilibrium demands, it easily follows that firm 2 will charge a higher price and enjoy a larger market share if 2*s > d*.

### The determination of the optimal *s*

In the first stage, firm 2 chooses the level of the unhealthy attribute that maximizes its profits. From now on, we will assume that *C*(*s*) = 0.5*cs*^2 ^with *c *∈ (0, 1]. By taking into account the equilibrium prices in the second stage of the game, we can write firm 2's profits as:(5)

When *s *≥ 2*d*, firm 2 serves the entire market, and its revenues are *s - d*. When *s *< 2*d*, firm 2 sells to consumers with degrees of guilt below . From (4),

it follows that its revenues are .

Let *d >*0.5. In this case, firm 2's profit function is given by . The optimal level of the unhealthy attribute s* satisfies . Taking the derivative of the profit function with respect to *s *yields:

It follows that  if 2 <*d*(9*c *- 2) and s* = 1 otherwise. When *d *is relatively high, we have maximal product differentiation for values of *c *that are relatively low. For high values of *c*, the level of the unhealthy attribute is below the highest feasible value, and product differentiation is less than maximal. As the unhealthy attribute is costly to produce and emotional costs are too high, firm 2 optimally chooses a level of the unhealthy attribute below 1.

**Proposition 2 ***Let d >*0.5. *The optimal level of the unhealthy attribute is:*

When *d >*0.5, once we take into account the optimal selection of the unhealthy attribute, we have that firm 2 will charge a higher price and enjoy a larger market share unless the condition 6 < 9*dc *is satisfied. Notice that this will happen when the emotional impact on utility derived from consuming the unhealthy good (*d*) and the parameter *c *of the cost function of such a good are relatively high.

Let *d *≤ 0.5. In this case, firm 2's profit function is given by (5). Firm 2 can choose the level of *s *that solves:

We can easily verify that the objective function is strictly increasing for all *s*. Thus, firm 2 would choose the highest value of *s*, e.g. *s *= 1, and its profits would be Π_2 _(1) = 1 - *d *- 0.5*c*. Alternatively, firm 2 can choose the level of *s *that solves:

It follows that the solution to this problem is  if 1 < 3*dc*. If 1 ≥ 3*dc*, the objective function is strictly increasing for all *s *and the optimal level of the unhealthy attribute is s* = 1, as firm 2's profits are higher for *s *≥ 2*d:*

For 1 < 3*dc*, we have two candidates for the optimal level of *s*: either s* = 1 or . By plugging into the profit function  we find that . By comparing firm 2's profits for the two candidates, we obtain:(6)

A sufficient condition for the numerator of (6) to be positive is that 10*d*^2 ^- 1 + 9*d *≥ 0. But this condition is always satisfied as  in the region we are considering. Therefore, firm 2 optimally chooses  when 1 < 3*dc *and *d *≤ 0.5.

**Proposition 3 ***Let d *≤ 0.5. *The optimal level of the unhealthy attribute is:*

When *d >*0.5, once we take into account the optimal selection of the unhealthy attribute, we have that firm 2 will charge a higher price and enjoy a larger market share for all values of *d *and *c*.

**Figure 1:**
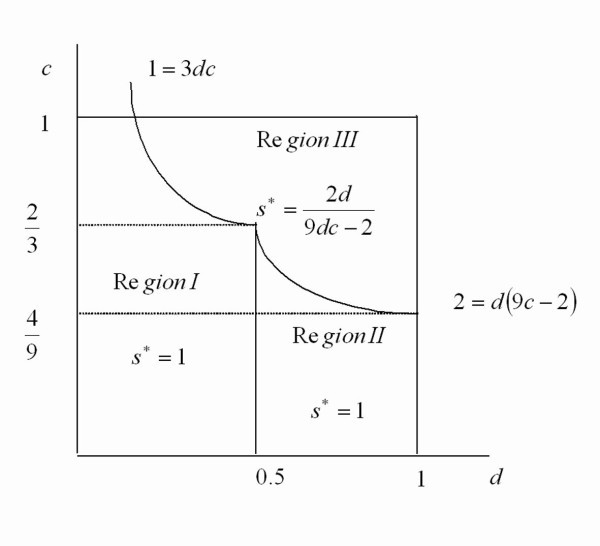
**The optimal level of the unhealthy attribute**.

The optimal level of the unhealthy attribute is depicted in Figure 1 below.

As we can see, the parameter space is divided into three regions. In region *I*, the only firm in the market is firm 2. It chooses the maximum feasible level of the unhealthy attribute. This holds for all values of *c *and . When , firm 2 chooses optimally the highest level of the unhealthy attribute as long as the cost *c *is relatively low . In region *II*, both firms are in the market. Firm 2 chooses the maximum level of product differentiation. Note that in this region, *d *is relatively high (above 0.5) and the parameter *c *is relatively low . Although the emotional/health cost *d *is high, the 9 low cost *c *still makes it profitable for the firm to choose the highest feasible level of the unhealthy attribute. In region *III*, the parameter *c *is relatively high. Consequently, it is optimal for firm 2 to choose a level of the unhealthy attribute below 1. In this region, for a given *d*, the optimal level of the unhealthy attribute diminishes with *c*. Likewise, for a given *c*, the optimal level of the unhealthy attribute diminishes with *d*.

### Welfare analysis and public policy

Social welfare is defined as the sum of consumers' surplus and firms' profits minus the production cost of the unhealthy attribute and the health costs. From a social perspective, the negative emotional impact on consumers' utility derived from consuming unhealthy products do not enter the social welfare function. Thus, social welfare is given by:

Let *d >*0.5. From (4) and Proposition 2, it follows that, in equilibrium, social welfare is:

We are interested in analyzing how social welfare behaves with *d*. When 2 <*d*(9*c *- 2), we have:

Thus, if 2 - 3*l <*0, the higher *d*, the larger social welfare is. So, from a social perspective, policies that increase the emotional costs from unhealthy consumption are desirable. We need sufficiently large health costs. On the other hand, if 2 - 3*l *≥ 0, it would be convenient to implement policies that reduce the emotional costs.

When 2 ≥ *d*(9*c *- 2), it follows that , and policies that reduce emotional costs are unambiguously desirable

Let *d *≥ 0.5. From (4) and Proposition 3, it follows that, in equilibrium, social welfare is:

Social welfare does not depend on *d *when 1 ≥ 3*dc*. Thus, public policies that change *d *do not have any effect on social welfare. When 1 < 3*dc*, we have the same result than before for the case 2 <*d*(9*c *- 2). The following propositions summarise the results.

**Proposition 4 ***Let d >*0.5. *When *2 <*d*(9*c *- 2), *public policies that increase the emotional costs are socially desirable if and only if *2 - 3*l *< 0. *If *2 ≥ *d*(9*c *- 2), *public policies that reduce emotional costs are unambiguously required*.

**Proposition 5 ***Let d *≤ 0.5. *When *1 < 3*dc*, *public policies that increase the emotional costs are socially desirable if and only if *2 - 3*l <*0. *When *1 ≥ 3*dc*, *public policies that influence d do not have any impact on social welfare*.

It has been argued that, from a social perspective, unhealthy consumption should be discouraged. However, consumers enjoy consuming unhealthy products, although they may be unaware of its negative health effects. At the same time, as consumers are willing to pay higher prices for unhealthy products, firms will be interested in manufacturing products with higher levels of unhealthy attributes. When all elements are taken into account, it is not clear which public policy is the best. According to the results of the model, unhealthy consumption should be discouraged when health social costs are sufficiently high. It is also required *d *and *c *to be sufficiently high. In this case, policies that increase the emotional costs *d *are unambiguously desirable. Counterintuively, when *c *is relatively low, the best policy is to reduce the emotional costs. When *d *is quite low , acting on *d *does not have any effect in welfare. For , increasing *d *is socially desirable only if *c *and *l *are sufficiently high. Otherwise, social welfare does not depend on *d*. Thus, we must be very careful about public policy recommendations, as the final effects can be the opposite to those pursued.

So far, we have focused on social welfare. However, in the real world, health authorities are mainly concerned with the intake of unhealthy food. In the context of the model, the aggregate consumption of the unhealthy attribute is given by  and health authorities attempt to reduce this consumption. As consumption of unhealthy products generates a negative externality, fiscal policies that levy taxes on unhealthy products have been proposed. These policies would reduce firm 2's sales and the level of *s*. These policies have been criticized as a moderate consumption of unhealthy food does not necessarily have pernicious effects on health (see Cash et al.(2005)). Leaving aside fiscal policies, health authorities can educate consumers through publicity campaigns emphasizing the negative effects derived from unhealthy consumption. These campaigns are designed to promote healthy consumption habits by making more costly (in emotional terms) to consume unhealthy products. In our model, these campaigns would increase the parameter *d*. It is assumed that publicity campaigns are fully effective in affecting the emotional costs. When emotional costs are low (region *I*), policies that marginally increase *d *do not have any impact on both s* and firm 2's sales as they do not depend on *d*. A non-marginal increase in *d *(to move to region *II *or *III*) is needed to reduce the aggregate consumption of the unhealthy attribute.

In region *II*, the aggregate consumption of the unhealthy attribute is given by firm 2's sales as s*(*d, c*) is equal to 1. From (4), firm 2's sales are , which clearly diminishes with *d*.

In region *III*, firm 2's sales are:

and the aggregate consumption of the unhealthy attribute is given by:

It can be easily checked that the higher *d*, the lower the aggregate consumption of the unhealthy attribute. In this case, both s*(*d, c*) and  are lowered with *d*. Thus, public policies that increase the emotional costs of unhealthy consumption are desirable.

## Conclusions

In this paper, we have carried out a theoretical analysis of the strategic choice made by firms regarding the type of food they market when they face consumers who not only care for the healthy/unhealthy attributes of the product but also experience emotional/health negative feelings when the food they consume has unhealthy characteristics. In particular, we have considered a duopolistic mode of vertical product differentiation to explore the decisions made by the firms regarding the type of food they produce and its consequences on prices and market shares. To simplify the analysis, we have assumed that one firm produces a healthy product while the other firm has to decide the degree of the unhealthy attribute in its product. Once the characteristics of the unhealthy product are fixed, firms compete in prices and choose simultaneously their prices. We find that, depending on the parameters of the model, product differentiation can be maximal or less than maximal. Maximal product differentiation takes place when the production costs of the unhealthy attribute are relative low. When these costs get higher, maximal product differentiation can still take place, but consumers must have a relatively low emotional/health cost. For sufficiently high costs, the degree of product differentiation is less than maximal. The firm that produces the unhealthy food charges a higher price and obtains a higher share of the market for almost all the feasible values of the exogenous parameters.

We have also explored the kind of public policies that can be undertaken in order to reduce the aggregate consumption of the unhealthy attribute. Besides fiscal policies that increase the price of the unhealthy food and discourage its consumption, educational campaigns have been considered. In the context of the model, these informational campaigns affect the emotional/health costs that consumers experience when they consume the unhealthy product. As was expected, we find that, depending on the parameters of the model, it is valuable to carry out these campaigns as they may reduce the demand of the unhealthy food as well as the degree of the unhealthy attribute. However, these policies do not have any effect when the emotional/health costs are relatively low. It remains an empirical question to estimate the value of these costs to determine whether educational campaigns may have a negative effect in the consumption of the unhealthy food.

The model, as stated, is highly stylized, and can be extended in several directions. Although mentioned, we have not analyzed public policies based on fiscal instruments to influence the production of the unhealthy product. Likewise, the description of consumers' preferences could be modified to include horizontal as well as vertical elements. After all, some attributes of food can be conceptualized as having horizontal features, while others (taste, quality, etc.) are vertical in their nature. The static nature of the model does not allow one to analyze long-term effects on health derived from unhealthy consumption habits. From the perspective of health authorities, this issue is important as health expenditures have increased considerably in developed countries during the last decade. A growing proportion of health expenditures is dedicated to treat health problems related to obesity. Thus, an interesting extension of the model would be to analyze, in a dynamic context, decisions for food production and consumption when there are future negative health effects. In this sense, the addictive component of the unhealthy food as well as self-control and time inconsistent preferences on the demand side should be taken into account. We hope to explore some of these issues in further research.

## Competing interests

The authors declare that they have no competing interests.

## Authors' contributions

RR-I carried out the model and the analytical steps. FA conceived of the study and participated in its designs. All authors drafted, read and approved the final manuscript.

## Endnotes

^a^See [12,13] and [14] for standard product differentiation models.

^b^Note that firm 1 will never choose a price above 1 as its demand would be zero.

^c^We can eliminate this equilibrium by restricting the parametric space for *d*. If *d *∈ (0.5, 1], this equilibrium is not feasible as 2*d *- *s *> 0 for all feasible values of *d *and *s*.
